# The Effects of *Loranthus parasiticus* on Scopolamine-Induced Memory Impairment in Mice

**DOI:** 10.1155/2014/860180

**Published:** 2014-06-17

**Authors:** Jin Bae Weon, Jiwoo Lee, Min Rye Eom, Youn Sik Jung, Choong Je Ma

**Affiliations:** ^1^Department of Medical Biomaterials Engineering, College of Biomedical Science, Kangwon National University, Hyoja 2-Dong, Chuncheon 200-701, Republic of Korea; ^2^Research Institute of Biotechnology, Kangwon National University, Chuncheon 200-701, Republic of Korea

## Abstract

This study is undertaken to evaluate cognitive enhancing effect and neuroprotective effect of *Loranthus parasiticus*. Cognitive enhancing effect of *Loranthus parasiticus* was investigated on scopolamine-induced amnesia model in Morris water maze test and passive avoidance test. We also examined the neuroprotective effect on glutamate-induced cell death in HT22 cells by MTT assay. These results of Morris water maze test and passive avoidance test indicated that 10 and 50 mg/kg of *Loranthus parasiticus* reversed scopolamine-induced memory deficits. *Loranthus parasiticus* also protected against glutamate-induced cytotoxicity in HT22 cells. As a result of *in vitro* test for elucidating possible mechanism, *Loranthus parasiticus* inhibited AChE activity, ROS production, and Ca^2+^ accumulation. *Loranthus parasiticus* showed memory enhancing effect and neuroprotective effect and these effects may be related to inhibition of AChE activity, ROS level, and Ca^2+^ influx.

## 1. Introduction

Alzheimer's disease (AD), the most common type of dementia, is progressive neurodegenerative disorder that results in memory impairment and cognitive dysfunction [1]. Age-related changes in the brain and genetics environmental and lifestyle factors cause AD generally.

The scientific pathogenesis of AD includes accumulation of amyloid-*β* plaque and neurofibrillary tangles, death of neuronal cell by oxidative stress, and cholinergic hypofunction [2–5]. Amyloid-*β* plaque and neurofibrillary tangles are found on areas of brain, including hippocampus and cortex of patient's brain. Acetylcholine (ACh), one of neurotransmitters, breaks down the ACh into choline and acetic acid and was dramatically decreased in AD brains. AChE inhibitors including Physostigmine, Tacrine, and Donepezil were commonly used for treatment of AD recently [6].

*Loranthus parasiticus *(Loranthaceae family) has been used for cholesterol lowing, Diuretic action, antibacterial effect, and antivirus effect.

*Loranthus parasiticus *has been recently reported to protect NG108-15 cells (a neuroblastoma/glioma hybrid cell line) against H_2_O_2_-induced oxidative damage and has antioxidant activity [7].

*Loranthus parasiticus* consisted of triterpene, lectin, polysaccharide, and alkaloid. Among the bioactive compounds, (+)-catechin exhibited neuroprotective effect in NG108-15 cells [8].

*Loranthus parasiticus* and (+)-catechin, the bioactive compound in* Loranthus parasiticus*, have been proven to protect NG 108-15 cells, neuroblastoma/glioma hydrid cell line. This result has suggested that* Loranthus parasiticus* may have cognitive enhancing effect.

Based on this, we evaluated cognitive enhancing effect of* Loranthus parasiticus* on mice subjected to amnesia. Morris water maze test and passive avoidance test are general behavior tests which are widely used to investigate memory and learning function of mice [9]. Scopolamine, muscarinic cholinergic receptor antagonist is known to induce memory impairment by administration in behavior tests [10].

Additionally, neuroprotective effect of* Loranthus parasiticus* in hippocampal neuronal cell line, HT22, was investigated. HT22 cells immortalized mouse hippocampal cell line is valuable for studying cellular mechanism of memory deficits associated with AD [11]. Glutamate is excitatory neurotransmitter and high concentration of glutamate-induced cell death by oxidative stress [12].

Additionally, we evaluated AChE inhibition, ROS generation, and Ca^2+^ accumulation to elucidate possible mechanism of effect of* Loranthus parasiticus*.

## 2. Materials and Methods

### 2.1. Plant Material and Extract Preparation

*Loranthus parasiticus* Merr. (Sang-ki-saeng) is purchased from Kyungdongmart (Seoul, Korea) and authenticated by Dr. Young Bae Seo, a professor of the College of Oriental Medicine, Daejeon University (Daejeon, Korea). Voucher specimens were deposited at the Kangwon National University in Chuncheon, Korea (no. CJ004M).

The steams of* Loranthus parasiticus* (600 g) are extracted with 80% methanol three times for 90 min by ultrasonics extracting device. The extract (56.43 g) was evaporated and freeze- dried to obtain powder.

### 2.2. Chemical Materials

Scopolamine, dimethyl sulfoxide, Trolox, carboxy methyl cellulose sodium (CMC), acetylcholine (ACh), acetylcholinesterase (AChE), 5,5-dithiobis-2-nitrobenzoic acid (DTNB), and glutamate were purchased from Sigma (St. Luis, Mo, USA). Donepezil was provided from Samjin Pharmaceutical Co., Ltd. (Seoul, Korea).

### 2.3. Animals

Male ICR mice (4-week old, 25–30 g) were purchased from Dae Han Bio Link Co., Ltd. (Chunkbuk, Korea) and used in Morris water maze test and passive avoidance test.

Mice were housed 7 per cage and maintained in temperature 20 ± 3°C under a 12/12 h light-dark cycle with access to feed and water* ad libitum *(commercial pellet).

All procedures of animal experiments were conducted according to guidelines of Kangwon National University IACUC (KIACUC). This study was approved by the ethics committee of the Kangwon National University.

### 2.4. Drug Administration

Powder of* Loranthus parasiticus *and donepezil were suspended in 0.5% carboxy methyl cellulose sodium (CMC). Scopolamine was dissolved in normal saline.

The mice were divided into six groups (*n* = 7) of test. Control group and scopolamine alone treated group were administered with 0.5% CMC. Donepezil (1 mg/kg) and three concentration groups (10, 50, and 100 mg/kg) of* Loranthus parasiticus* were administered orally. After 90 min, scopolamine was subcutaneously administrated except control group. After 30 min, the water maze test and passive avoidance test were performed.

### 2.5. Morris Water Maze Test

Morris water maze test was carried out as method previously described [9, 13]. The water maze was consisted of large circle pool and was filled to a depth of 30 cm with opaque water with white milk. The water maze was divided into four equal quadrants. A white plastic platform was submerged in one of the quadrant of the pool, 1 cm below the surface of the water. In the following 4 days, mice were given two trials with interval of 15 min for four consecutive days. The time taken to find the hidden platform was recorded for trial test as escape latency (s). In the last day, mice received probe trial session which removed the platform for 60 s. Swimming time in target quadrant, where the platform was placed was recorded. Smart (ver. 2.5.21) video-tracking system is used to monitor and analyze all swimming activity of mice and record escape latency, swim distance, and speed.

### 2.6. Passive Avoidance Test

Passive avoidance test was performed according to previously described method [13, 14]. Passive avoidance apparatus consists of light compartments and dark compartments with an electrifiable grid floor. Training trial was performed on the first day. 24 h after the training trial, test trial was performed and mice were again placed in the light compartment. Time taken to enter the dark compartment was recorded up to 180 s during each trial as the latency. If a mouse waited more than 180 s in the light compartment, the mouse was excluded from experiment.

### 2.7. AChE Activity Assay

AChE activity was determined using a spectrophotometric method, as described by Ellman et al. coupled enzyme assay [15]. The reaction mixture that contained 33.3 *μ*L of 4% DTNB, 50 *μ*L of various concentrations of* Loranthus parasiticus*, and 16.7 *μ*L of AChE (4.3 unit/mL) was preincubated for 5 min at 37°C, and then 50 *μ*L of ACh was added to the reaction mixture. After incubation for 3 min, the optical density value was measured at 412 nm.

### 2.8. Cell Viability

HT22 cells, a neuronal cell line derived from mouse hippocampus, were used widely to study mechanisms of glutamate-induced cell death [11]. HT22 cell lines were cultured in DMEM supplemented with 10% (v/v) fetal bovine serum (FBS), 1% penicillin/streptomycin, NaHCO_3_ (2 mg/mL), and 15 mM HEPES and were maintained at 37°C at humidified 5% CO_2_.

Cell viability was determined by MTT assay by described previous method. Cultured HT22 cells were seed at 6.7 × 10^4^ cell/well in 48-well plate and incubated for 24 h at 37°C at 5% CO_2_. After cells were treated with 1, 10, and 100 *μ*g/mL of* Loranthus parasiticus *and 50 *μ*g/mL of Trolox (positive control) for 1 h, glutamate (2 mM) was added and incubated for 24 h. After incubation, 1 mg/mL of MTT solution was added to each well for 3 h. MTT formazan was dissolved by dimethyl sulfoxide (DMSO) and the optical density was measured at 570 nm using an ELISA reader.

### 2.9. Measurement of ROS

ROS level was measured using the dye DCF-DA (2′7′-dichlorofluorescein diacetate) [16]. The HT22 cells were treated with 1, 10, and 100 *μ*g/mL of* Loranthus parasiticus* and 50 *μ*g/mL of Trolox and 2 mM of glutamate for 8 h. After incubation, 10 *μ*M DCF-DA was added to the cells and then incubated at 37°C for 30 min. The culture medium was removed and then washed twice with PBS. The intensity of fluorescence was detected at an excitation wavelength of 490 nm and emission wavelength of 525 nm.

### 2.10. Calcium Measurement

The intracellular Ca^2+^ concentration in HT22 cells was detected by using the Fura-2, AM [17]. HT22 cells were plated in 48-well plates and treated with 1, 10, and 100 *μ*g/mL of* Loranthus parasiticus*, 50 *μ*g/mL of Trolox, and 2 *μ*M Fura-AM. After 1 h treatment, glutamate was added to each well for 2 h. Cells were washed with PBS and then suspend in 1% Triton X-100. Fluorescence of Fura-2, AM was measured at an excitation wavelength of 400 nm and emission wavelength of 535 nm.

### 2.11. Statistics

All the results were expressed as means ± S.E.M. In probe trial of Morris water maze test and passive avoidance test and cell experiments, the results were analyzed with one-way analysis of variance (ANOVA) followed by Tukey's post hoc test. Escape latency in the Morris water maze test was analyzed using two-way ANOVA followed by Tukey's analysis. The results were considered as statistical significance at *P* < 0.05.

## 3. Results

### 3.1. Effect of* Loranthus parasiticus* on Morris Water Maze Test

**Figure 1:**
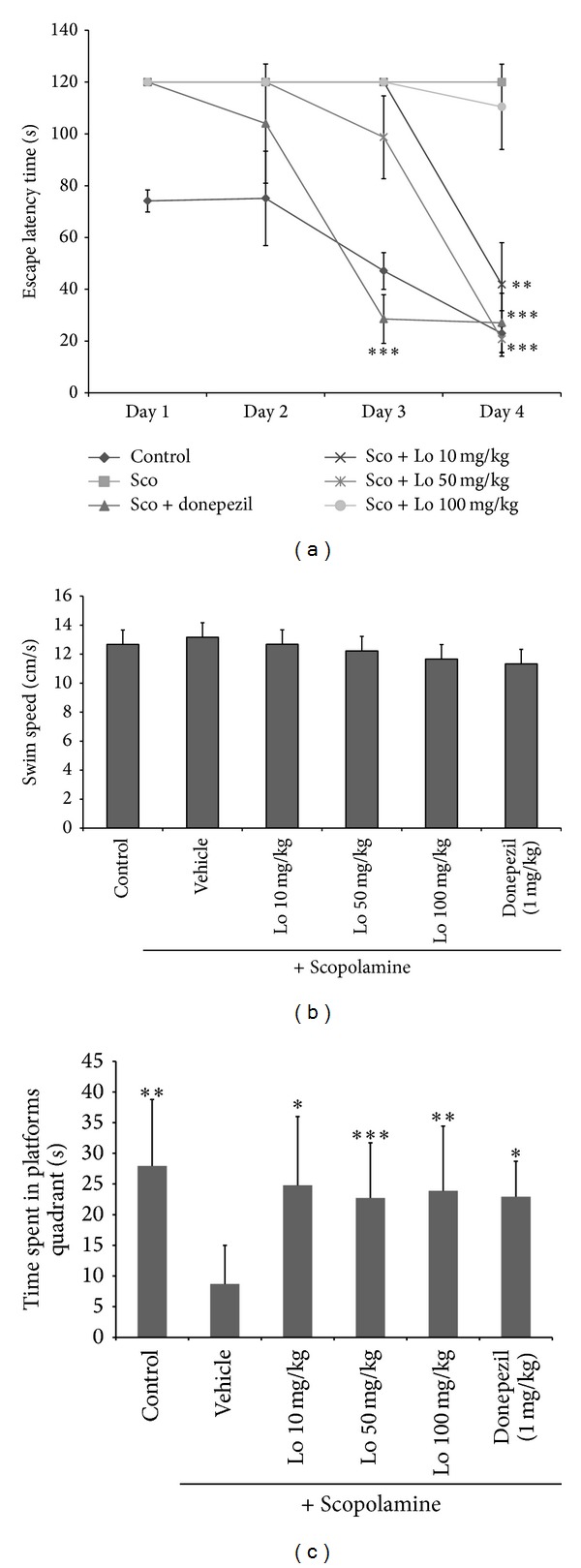
(a) Effect of* Loranthus parasiticus* on escape latency (s) of 4 day-trial in Morris water maze test.* Loranthus parasiticus* (10, 50, and 100 mg/kg body weight, P.O.) and donepezil group (positive control; 1 mg/kg body weight, P.O.) treated 90 min before scopolamine administration. Scopolamine treated 30 min before water maze test. The values shown are the mean escape latency ± SD (*P < 0.05, **P < 0.01, and ***P < 0.001 versus the scopolamine group). Control: control group, Sco: only scopolamine-treated group, and Sco + donpezil: scopolamine and donepezil treated group. Sco + Lo: scopolamine and* Loranthus parasiticus* treated group. (b) Swim speed of mice in Morris water maze test. Data represent means ± S.E.M. Control: control group, vehicle: only scopolamine-treated group, and donpezil: scopolamine and donepezil treated group. Lo: scopolamine and* Loranthus parasiticus* treated group. (c) Effect of* Loranthus parasiticus* (10, 50, and 100 mg/kg) on probe trial. Control: control group, vehicle: only scopolamine-treated group, and donpezil: scopolamine and donepezil treated group. Lo: scopolamine and* Loranthus parasiticus* treated group. Data represent means ± S.E.M. (*P < 0.05, **P < 0.01, and ***P < 0.001 versus the scopolamine group).

Scopolamine, a muscarinic acetylcholine receptors antagonist, has been shown to impair memory. Scopolamine-treated group exhibited escape latency throughout the 4 training days longer than control group. 50 mg/kg of* Loranthus parasiticus* significantly decreased the escape latency time of mice prolonged by scopolamine administration on the 3rd and 4th days (*F* (1, 64) = 3.99, *P* < 0.05). Donepezil, positive control, also significantly reduced escape latency time. Water maze test exhibited significant effects for treatment groups (*F* (5, 192) = 2.26, *P* < 0.001) and test days (*F* (3, 192) = 2.06, *P* < 0.001) (Figure 1(a)).

There was no significant difference on average swimming speed of mice between the groups during test trial for 4 days (Figure 1(b)). The result indicated that locomotor activity of mice had no effect on the escape latency of trials.

In probe trial, scopolamine-treated group significantly showed swimming time in the target quadrant shorter than control group.* Loranthus parasiticus *treated group significantly increased swimming time in the target quadrant compared with scopolamine-treated group (Figure 1(c)).

### 3.2. Effect of* Loranthus parasiticus *on Passive Avoidance Test

**Figure 2:**
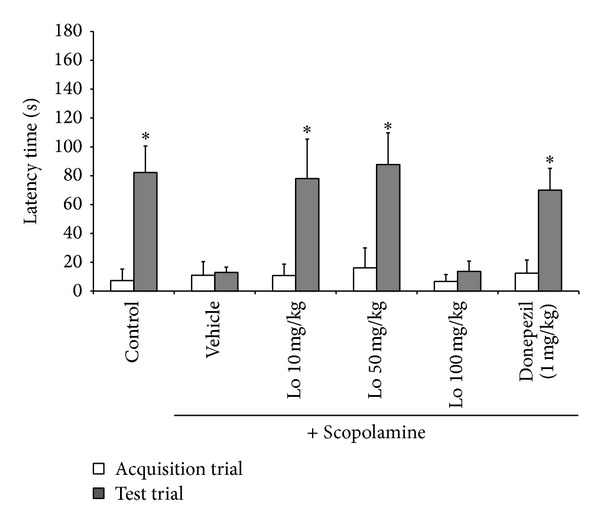
Effect of* Loranthus parasiticus* (10, 50, and 100 mg/kg) on scopolamine-induced memory impairment in the passive avoidance test. Control: control group, vehicle: only scopolamine-treated group, and donpezil: scopolamine and donepezil treated group. Lo: scopolamine and* Loranthus parasiticus* treated group. Mean latency time (s) ± SD (*n* = 7) *P < 0.05, **P < 0.01, and ***P < 0.001 compared with the scopolamine group.

In acquisition trial, latency time had no significant difference between all experiment groups (*F* (5, 24) = 2.62, *P* = 0.619). Scopolamine-treated group decreased latency time compared with control group. However, the shorten latency time was significantly reversed by 10 (*F* (1, 8) = 5.32, *P* < 0.05) and 50 mg/kg (*F* (1, 8) = 5.32, *P* < 0.05) of* Loranthus parasiticus* treatment, as compared to the scopolamine-treated group. Treatment with donepezil, as positive control, also significantly increased latency time shortened by scopolamine (*F* (1, 8) = 5.32, *P* < 0.05) (Figure 2).

### 3.3. Effect of* Loranthus parasiticus *on Acetylcholinesterase (AChE) Activity

We assessed the AChE activity to confirm whether effect of* Loranthus parasiticus* in Morris water maze test and passive avoidance test is associated with inhibition of AChE activity.* Loranthus parasiticus* inhibited AChE activity with an IC_50_ value of 1542.6 *μ*g/mL. The IC_50_ donepezil (positive control) also inhibited AchE activity (IC_50_ value: 12.6 *μ*g/mL).

### 3.4. Effect of* Loranthus parasiticus *on Glutamate-Induced Cytotoxicity in HT22 Cells

**Figure 3:**
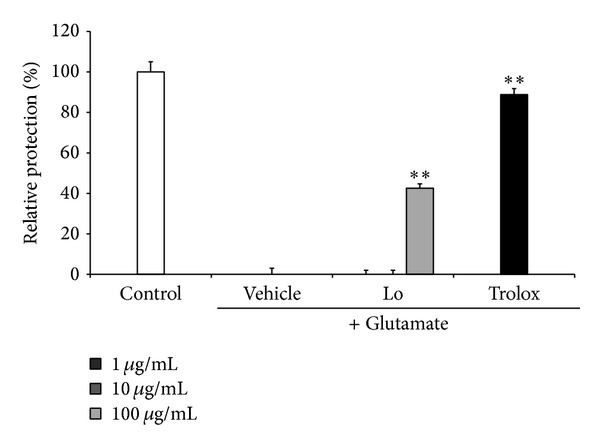
Effect of* Loranthus parasiticus* (1, 10, and 100 *μ*g/mL) on glutamate-induced cell death in HT22 cells. Control: control group, vehicle: only glutamate treated group, and Trolox: glutamate and Trolox treated group. Lo: glutamate and* Loranthus parasiticus* treated group. Data represent means ± S.E.M. (*P < 0.05, **P < 0.01, and ***P < 0.001 versus the glutamate treated group).

We performed MTT assay to investigate neuroprotective effect of* Loranthus parasiticus *on glutamate-induced cell death in HT22 cells. We found that 2 mM glutamate treatment reduced cell viability.* Loranthus parasiticus* significantly improves cell viability against glutamate-induced cytotoxicity.* Loranthus parasiticus* exhibited 42.55 ± 8.67% of relative protection at concentrion of 100 *μ*g/mL against glutamate-induced cell death (Figure 3). Trolox, as positive control, also significantly protects HT22 cells against glutamate-induced cytotoxicity.

### 3.5. Effect of* Loranthus parasiticus *on ROS Production and Intracellular Ca^2+^ Concentration

**Figure 4:**
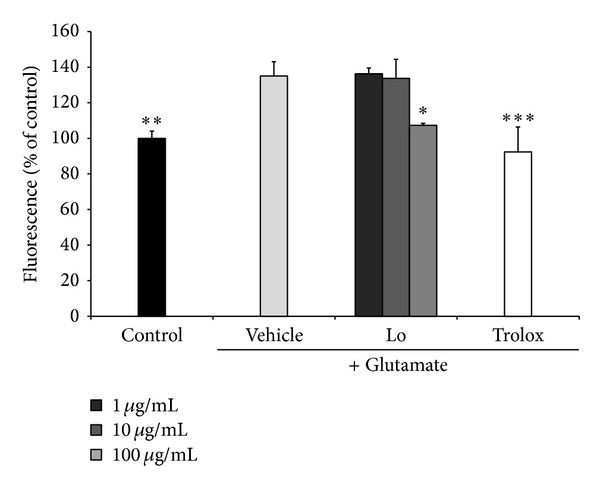
The effect of* Loranthus parasiticus* (1, 10 and 100 *μ*g/mL) on glutamate-induced ROS generation in HT22 cells. Control: control group, vehicle: only glutamate treated group, and Trolox: glutamate and Trolox treated group. Lo: glutamate and* Loranthus parasiticus* treated group. Data represent means ± S.E.M. (*P < 0.05, **P < 0.01, and ***P < 0.001 versus the glutamate treated group).

We evaluated effect of* Loranthus parasiticus* on ROS generation by fluorescence using dye DCF-DA. Treatment of HT22 cells with glutamate led to increase in ROS production (135.06 ± 8.05%) compared with control cells. Pretreatment of cells with* Loranthus parasiticus* (100 *μ*g/mL) significantly reduced ROS generation (107.30 ± 1.08%, *P* < 0.05) (Figure 4).

**Figure 5:**
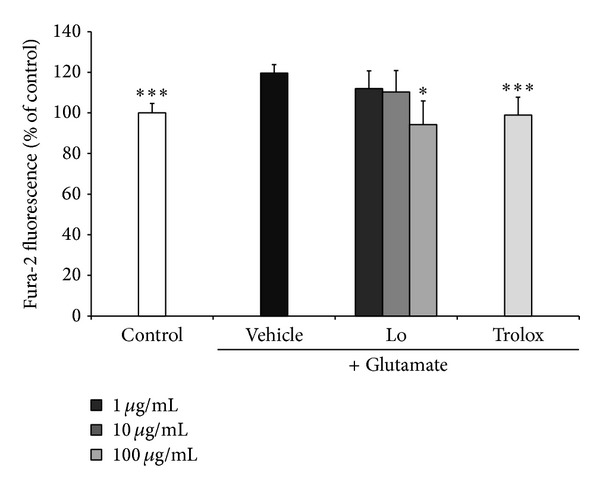
The effect of* Loranthus parasiticus* (1, 10, and 100 *μ*g/mL) on glutamate-induced Ca^2+^ influx in HT22 cells. Control: control group, vehicle: only glutamate treated group, and Trolox: glutamate and Trolox treated group. Lo: glutamate and* Loranthus parasiticus* treated group. Data represent means ± S.E.M. (*P < 0.05, **P < 0.01, and ***P < 0.001 versus the glutamate treated group).

We assessed the Ca^2+^ level using the Fura-AM to check whether* Loranthus parasiticus* can decrease intracellular Ca^2+^ concentration in HT22 cells. Ca^2+^ concentration was increased in cells treated with glutamate (119.63 ± 4.11%). However, Ca^2+^ concentration in cells pretreated with* Loranthus parasiticus* (100 *μ*g/mL) significantly decreased compared with glutamate-treated cells (94.23 ± 8.80, *P* < 0.05) (Figure 5).

## 4. Discussion

In the present study, we found that* Loranthus parasiticus* ameliorated scopolamine-induced memory impairment in mice by decrease of AChE activity.* Loranthus parasiticus* showed protective effect against glutamate-induced cell death by inhibition of ROS and intercellular Ca^2+^ concentration on HT22 cells.

Cognitive enhancing effect of* Loranthus parasiticus* was evaluated using Morris water maze test and passive avoidance test. Scopolamine induced memory dysfunction in mice by administration. Two main types of ACh receptors are muscarinic and nicotinic receptors [18]. Scopolamine inhibited the cholinergic neurotransmission Ach* via* blocking muscarinic receptors [3, 19].

Morris water maze test is one of the most widely used behavioral tests for studying hippocampus-dependent spatial memory and leaning. We designed the Morris water maze test to measure effect of* Loranthus parasiticus* on scopolamine-induced memory deficits in mice [9]. In Morris water maze test, parameters include escape latency time taken to locate the platform of mice in trial session for test day and time spent in platform quadrant in probe trial were investigated and these parameters represent learning and memory function of mice.

*Loranthus parasiticus* significantly reduced escape latency increased by scopolamine administration and increased swim time in target quadrant compared to scopolamine-treated group.

Passive avoidance test is a fear-aggravated test performed to access memory function. Passive avoidance test was used to assess memory function based on the natural tendency of animals formed between an aversive stimuli [20, 21]. In this study, subjects who learn to avoid aversive stimulus (foot shock) were described. Time which mice take to move from light compartment to dark compartment after exposure to foot shock was recorded as latency time.

*Loranthus parasiticus* significantly increased latency time taken for mice to move two compartments compared with scopolamine-treated group. These results of behavior tests indicated that* Loranthus parasiticus* significantly reversed scopolamine-induced memory impairment in the mice model.

10 and 50 mg/kg of* Loranthus parasiticus *treated group showed higher cognitive-enhancing effect than 100 mg/kg of* Loranthus parasiticus *treated group in the Morris water maze test and passive avoidance test.

It suggested that the effect of of* Loranthus parasiticus *was not due to increased concentration of* Loranthus parasiticus*. This replicates the result which has been shown with other previous studies [22, 23].

ACh is neurotransmitter and released ACh moves the gap between the synapses. AChE hydrolyzed the neurotransmitter ACh by cleaving acetylcholine into acetate and choline to continually produce ACh at cholinergic synapses [24]. However, high concentration of AChE decreased ACh level and was implicated in some neurodegenerative diseases including AD [25]. AChE activity is present in AD brain. Scopolamine induced memory impairment by increased AChE activity. In this study, scopolamine-treated group increased AChE activity but* Loranthus parasiticus* reversed the effect of scopolamine. It is suggested that possible mechanism of memory improving effect of* Loranthus parasiticus* may be associated with AChE activity in cholinergic system.

Neuronal cell death may play a role in the pathological of AD [26]. Cell loss in hippocampus results in loss of memory and learning function. HT22 cells are a subline derived from parent HT4 cells (originally immortalized from mouse primary hippocampal cell) and provide* in vitro* system to study mechanism of cell death by glutamate cytotoxicity, a cause of neurodegenerative disease such as AD.

Glutamate acts as endogenous excitatory neurotransmitter in the central nervous system (CNS). Excessive glutamate-mediated excitotoxicity causes prolonged stimulation of NMDA receptors and can lead to neuronal cell death [27]. Glutamate led to oxidative stress by inhibition of cysteine uptake through the cysteine/glutamate antiporter and increased intracellular ROS generation [28]. The increased ROS production medicated oxidative stress and was implicated in AD [11]. ROS production was caused through Ca^2+^-dependent mechanism. Glutamate involved Ca^2+^ influx into neuronal cell via N-methyl-D-aspartate (NMDA) receptors [29, 30]. Reactive oxygen species (ROS) generation and mitochondrial dysfunction occur as a result of intracellular Ca^2+^ accumulation [31].* Loranthus parasiticus* protected neuronal cell against glutamate-induced cell death on mouse hippocampal HT22 cells. In addition,* Loranthus parasiticus *inhibited ROS production and intracellular Ca^2+^ accumulation. These results indicated that inhibition of ROS and intracellular Ca^2+^ production of* Loranthus parasiticus* may be involved in the neuroprotective activity. Scopolamine-induced memory impairment is also associated with oxidative stress in hippocampus [32].

In conclusion*, Loranthus parasiticus* improves memory and learning function by decrease of AChE activity and decreased glutamate-induced neuronal cell death via antioxidant system and inhibition of ROS and Ca^2+^ level. Further studies are needed to elucidate the possible mechanism of effect of* Loranthus parasiticus*. Overall results of study suggested that* Loranthus parasiticus *has therapeutic potential for the prevention or treatment of neurodegenerative diseases.
